# Squeezing Droplet Formation in a Flow-Focusing Micro Cross-Junction

**DOI:** 10.3390/mi15030339

**Published:** 2024-02-28

**Authors:** Filippo Azzini, Beatrice Pulvirenti, Massimiliano Rossi, Gian Luca Morini

**Affiliations:** Department of Industrial Engineering, Alma Mater Studiorum Università di Bologna, Viale Risorgimento 2, 40136 Bologna, Italy; beatrice.pulvirenti@unibo.it (B.P.); massimiliano.rossi13@unibo.it (M.R.); gianluca.morini3@unibo.it (G.L.M.)

**Keywords:** two-phase flow, OpenFOAM, droplet simulation, micro-junction

## Abstract

Motivated by the increasing need of optimised micro-devices for droplet production in medical and biological applications, this paper introduces an integrated approach for the study of the liquid–liquid droplet creation in flow-focusing micro cross-junctions. The micro-junction considered is characterised by a restriction of the channels cross-sections in the junction, which has the function of focusing the flow in the region of the droplet formation. The problem is studied numerically in the OpenFOAM environment and validated by a comparison with experimental results obtained by high-speed camera images and micro-PIV measurements. The analysis of the forces acting on the dispersed phase during the droplet formation and the diameter of the droplets obtained numerically are considered for the development of a model of the droplet breakup under the squeezing regime. On the basis of energy balancing during the breakup, a relation between interfacial tension, the size of the cross-sections in the junction, and the time interval needed for droplet creation is obtained, which yields a novel correlation between the dimensionless length of the droplet and the dimensionless flow rate. This research expands our knowledge of the phenomenon of drop creation in micro-junctions with restrictions providing new aid for the optimal design of micro-drop generators.

## 1. Introduction

In recent years, the proliferation of micro-fabrication technologies has provided a broad range of microfluidic applications in engineering. Micro-droplet technology has proven to be a promising and flexible platform for microfluidic functions, such as the production of mono-disperse particles, droplets, bubbles, foams, and emulsions with precise control of components and sizes, which can give advancements in chemical, pharmacological, medical, and industrial applications. Many microfluidic devices have been designed to generate uniform droplets, including geometry-dominated devices [1], flow-focusing devices [2], T-junctions [3,4], and co-flowing devices [5]. However, the mechanism of droplet formation in micro-junctions is not yet fully understood. The two-phase flow characteristics are determined by flow conditions, fluid properties, and the geometry of the micro-device. The first key parameter in the control of the diameter of droplets created within a micro-junction is the flow rate ratio, i.e., the ratio between the flow rate of the dispersed phase to the flow rate of the continuous phase [6]. Other key parameters are the geometry of the junction and the properties of the two fluids, such as the capillary number or the viscosity ratio [7]. Several micro-junction geometries are presented in the literature, such as the most studied micro T-junctions [8] and the micro cross-junctions [9]. For these devices, the dependence of the two-phase flow patterns on the flow rate ratio has been shown [10], and correlations have been provided between the droplet diameter and important key parameters, such as the flow rate ratio and the capillary number [11]. The mechanism of droplet formation, called droplet breakup, has been largely studied for two decades on the micro-scale. Different mechanisms have been identified, such as squeezing, dripping and jetting [12]. By studying the underlying mechanisms in the droplet breakup, some scaling laws have been established to predict the size of droplets produced in micro-junctions. However, more experimental data are needed to generalise the results. Based on the statistical analysis of a large number of available literature data, Steegmans et al. [13] have shown that none of the scaling models, which are developed to predict droplet formation in a microfluidic T-junction, are general enough to describe the original data and data from other literature sources. Numerical approaches can be complementary to experimental investigation if validated and integrated with the experimental measurements. The aim of this work is the prediction of the droplet generation in a micro cross-junction under the squeezing regime. The real micro-device considered has a restriction in the junction, i.e., the diameter of the micro-channels in the junction is smaller than the diameters of the micro-channels out of the junction. The dynamics of the droplet creation within the micro-junction is studied numerically by means of a VOF based code in the OpenFOAM environment. The simulation results are compared with experimental data obtained on the real cross-junction by means of images obtained by a high-speed camera and micro-PIV measurements. The numerical results are used to build a model for the prediction of the droplet diameter, based on energy balancing during the breakup. A relation between the interfacial tension, size of neck cross-section, and the time interval needed for droplet creation is obtained. By this approach, droplet diameters are predicted. The results obtained by the model are in a very good agreement with the validated numerical simulations. This study can provide useful information for understanding micro-droplet dynamics, providing a basis for optimal design of multi-phase microfluidic devices. The design of micro-junctions with the aim of producing drops in a very narrow and reproducible range of diameters is currently based on the use of correlations that are not suitable for all the junctions and the experimental studies are not always available. This research contributes to micromachines providing new aid for the optimal design of micro-drops generators.

## 2. Materials and Methods

### 2.1. Numerical Simulations

**Figure 1:**
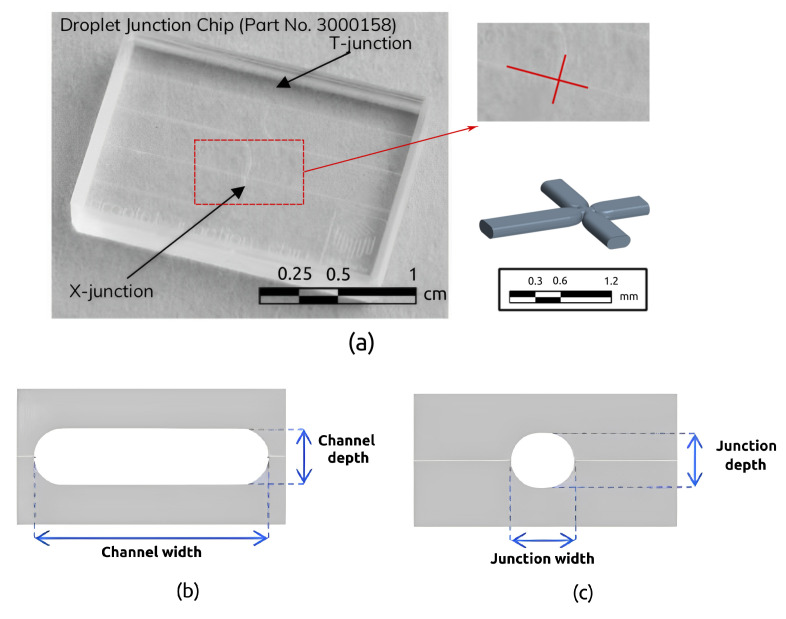
Dolomite cross-junction chip and 3D cad recostruction of the micro-junction (**a**) [14]. Channel section (**b**) and restriction section (**c**).

The micro-junction geometry considered for the numerical simulations refers to a real micro-device manufactured by Dolomite Microfluidics, shown in Figure 1 (top). The junction connects four micro-channels with a stadium-shaped cross-section, as shown in Figure 1 (bottom), with a restriction in the junction.

**Figure 2:**
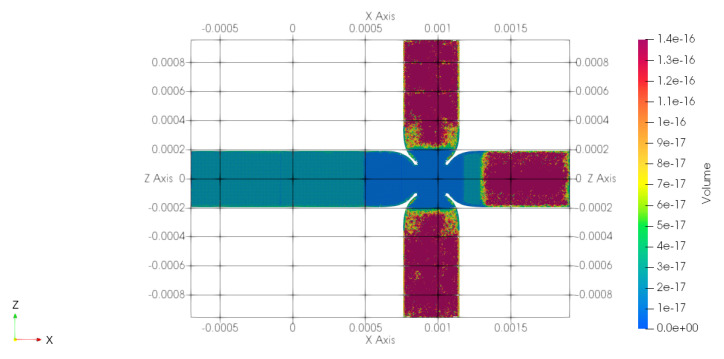
Top view of the polyhedral mesh.

The channel width is W=390.0 µm, while the channel depth is H=190.0 µm. At the junction, the restriction has a width of $W_{j}=195.0$ µm and a depth of $H_{j}=190.0$ µm. The channel aspect ratio is β=H/W=0.4871, while the aspect ratio in the junction is $\beta_{j}=H_{j}/W_{j}=0.974$. The restriction ratio, defined as the ratio between the channel width at the restriction and the channel width, is $\gamma =W_{j}/W=0.5$. For the numerical simulations, a computational domain, shown in Figure 1, has been built, characterised by three inlet branches with a length of $L_{in}=599.0$ µm, and one outlet branch with a length of $L_{out}=1299.0$ µm. The ratios between the inlet and outlet branches and the hydraulic diameter of the channels, $D_{h}=266.24$ µm, are $L_{in}/D_{h}=2.25$ and $L_{out}/D_{h}=4.88$, respectively. A polyhedral mesh has been built, Figure 2 shows the volume (in m^3^) of the cells, where the red zones at the three inlets are characterised by larger grid elements, while the outlet region is characterised by smaller elements, with a refinement in the junction where the breakup phenomena occur, and the total number of elements is about 5 million.

The solver used to perform the numerical simulations is the *interFoam* solver provided by the open-source code OpenFOAM. This is a solver for two incompressible, isothermal and immiscible fluids, based on a VOF (volume of fluid) phase-fraction-based interface capturing approach; similar methodologies have been extensively utilised in other works within the microfluidics field [15,16,17]. The equations solved are the continuity equation

(1) ∇·u=0

and the Navier–Stokes equation

(2) $$\frac{\partial (\rho u)}{\partial t}+\nabla \cdot \left(\rho \mathrm{uu}\right)=-\nabla p+\nabla \cdot \tau +\rho g+f_{\sigma }$$

plus an additional equation to capture the interface between the fluids, the advection of the scalar quantity α,

(3) $$\frac{\partial \alpha }{\partial t}+\nabla \cdot \left(\alpha u\right)=-\nabla \cdot \left[\alpha \left(1-\alpha \right)u_{r}\right]$$

The field α is used to distinguish the two fluids and is defined as

(4) $$\alpha =\left\{\begin{array}{cc} 1 & \mathrm{in}\;\mathrm{the}\;\mathrm{continuous}\;\mathrm{phase}\;\\ 0.5 & \mathrm{at}\;\mathrm{the}\;\mathrm{interface}\;\\ 0 & \mathrm{in}\;\mathrm{the}\;\mathrm{disperse}\;\mathrm{phase} \end{array}\right.$$

To solve these equations, the MULES (multi-dimensional limiter for explicit solution) algorithm is used in order to guarantee the boundedness of the solution and obtain more smeared interfaces. The last term of the right-hand side of Equation (3) is the so-called compression term, which is not negligible only at the interface between the two fluids. In this term, the relative velocity $u_{f}$ is present, which is defined as follows:

(5) $$u_{f}=min(C_{\alpha }\left|u|,max(|u|)\right)\frac{\nabla \alpha }{\left|\nabla \alpha \right|}$$

For micro-fluidic applications, $C_{\alpha }$ can be taken to be equal to 1 [18]. In Equation (2), the source term $f_{\sigma }$ is used to estimate the surface tension forces by means of the Continuum Surface Force (CSF) model, which gives

(6) $$f_{\sigma }=\sigma \kappa \nabla \alpha$$

where κ is the curvature, evaluated starting from the volume fraction α,

(7) $$\kappa =\frac{\nabla \alpha }{\left|\nabla \alpha \right|}$$

The dispersed phase is water, with density $\rho_{d}$ = 998 kg/m^3^ and viscosity $\nu_{d}$ = 8.788$\cdot 10^{-7}$ m^2^/s. The continuous phase is oil, with a density of $\rho_{c}$ = 950 kg/m^3^ and a viscosity of $\nu_{c}$ = 1.900$\cdot 10^{-5}$ m^2^/s.

The dispersed phase enters from the channel along the *x*-axis and the continuous phase enters from the two channels along the *z*-axis. For all the inlets, the mass flow rate is imposed, and in the outlet section, a fixed pressure (*p* = 0) is considered as the boundary condition. The dispersed phase flow rate has been varied in the range of 0.6–7.5 mL/h, while the continuous phase flow rate has been fixed to 7.5 mL/h. The regime is laminar, as the Reynolds number calculated for the dispersed phase is $Re_{c}=v_{c}D_{h}/\nu_{c}=0.3$, and $Re_{d}=v_{d}D_{h}/\nu_{d}$, ranges from 0.5 to 6. The capillary number for the dispersed phase, $Ca_{d}=\mu_{D}u_{d}/\sigma$ ranges from 0.00005 to 0.0006, where σ=0.04244 N/m is the surface tension between the two fluids [11]. The capillary number for the continuous phase is $Ca_{c}=\mu_{D}u_{d}/\sigma =0.0134$. Then, the regime for droplet creation is the squeezing regime for all the cases in these ranges [11].

**Figure 3:**
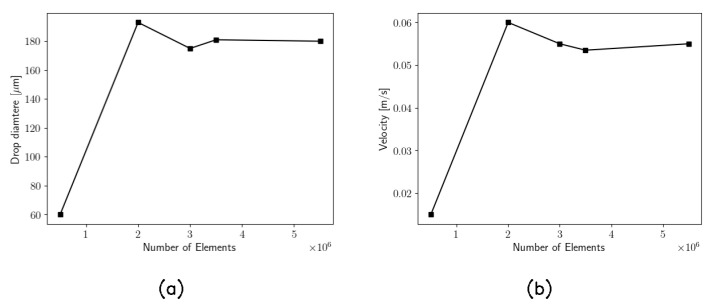
(**a**) Radius of the droplet; (**b**) asymptotic velocity of the droplet. These two quantities are calculated far from the junction, where these values become constant over time, reaching their asymptotic value.

Five meshes have been built to check the grid convergence. The convergence has been proven by comparing the velocity and the diameter of the droplets as a function of the number of elements, as shown by Figure 3.

### 2.2. Experimental Set-Up

**Figure 4:**
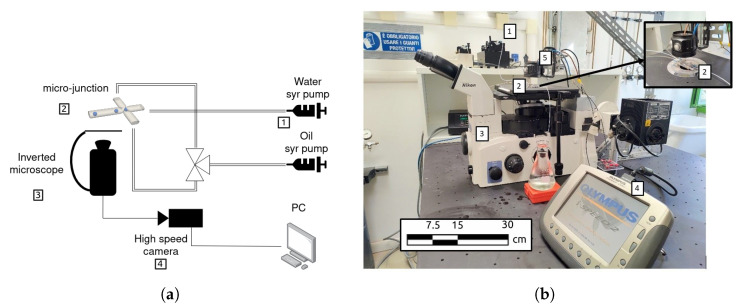
(**a**) Schematic and (**b**) picture of the experimental apparatus consisting of two syringe pumps (1) to control the flow rates of the working fluids and the microfluidic device (2) placed on an inverted microscope (3) connected to a high-speed camera (4).

An experimental set-up was designed and assembled to validate the numerical simulations. A schematic and a picture of the experimental apparatus are shown in Figure 4. Two syringe pumps (Harvard Instruments PHD 400) were used to control the flow rates of the two working fluids. The pumps were connected to the microfluidic chip with the micro-junction (Dolomite Microfluidics), which was placed over an inverted microscope (Nikon Eclipse TE2000-U) and observed through a 10× objective lens with NA = 0.25. High-speed video recordings of the droplets in the microfluidic device were taken using a high-speed camera (Olympus I-Speed I). The illumination was provided by a high-power LED powered by DC current. The shapes and dimensions of the droplets were determined by a custom-made image processing code developed in the Python environment. The two-dimensional velocity field in the mid-plane of the dispersed phase was measured utilising the micro-PIV technique [19]. For these measurements, polystyrene micro-spheres with a diameter of 1.19 µm and a density of 1050 kg/m^3^ were inserted at a low concentration in the dispersed phase and used as a passive tracer. The micro-PIV analysis was performed using the open-source library *DefocusTracker* in the MATLAB environment [20]. First, a pre-processing step was applied for background removal. The PIV analysis was performed with interrogation windows of 32 × 64 pixels with 50% overlap. The estimated thickness of the measurement planes in terms of depth of correlation [21] is equal to 20.4 µm.

## 3. Results

### 3.1. Validation of the Numerical Simulations

**Figure 5:**
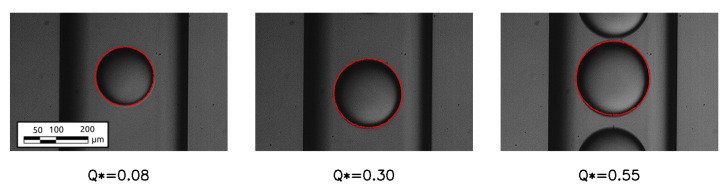
Superposition between the interface obtained numerically (red dots) and the images of the drops for different dimensionless flow rates.

In order to validate the simulations, the dimensions of the droplets obtained numerically were compared with the dimensions of the droplets obtained experimentally for the same working conditions. The shape and the diameter of the drop are taken far away from the junction, where they remain constant over time since the non-stationary effects due to breakup have been exhausted. The maximum error obtained, considering the entire range of cases studied (Table 1), is equal to 5%. A qualitative comparison for three of the considered cases is presented in Figure 5, showing an excellent agreement between numerical results and experiments.

**Figure 6:**
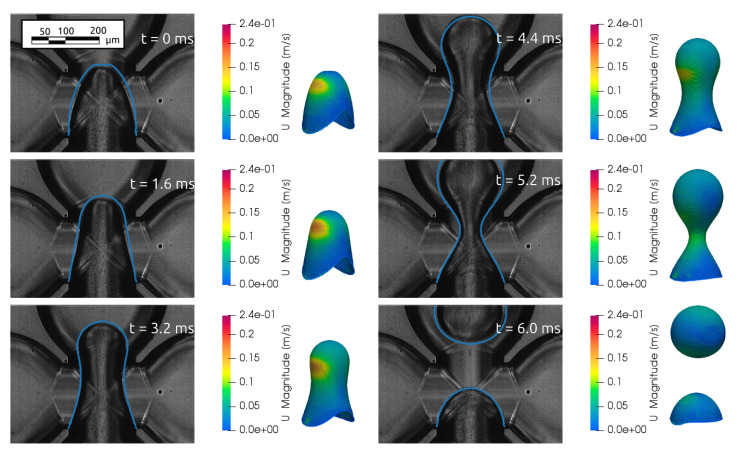
Comparison between the interface obtained experimentally and numerically (blue line) for the sequential moments leading to the breakup of the drop, with time expressed in milliseconds (ms), in the case with Q = 0.08.

Moreover, the dynamics of the breakup phenomena in the experiments were compared with the results of the simulations. Figure 6 shows the comparison of the numerical data and the experimental data for the case with the dimensionless flow rate, defined as a ratio between the dispersed phase and continuous phase equal to 0.08. The 3D shapes of the interface are coloured with the velocity magnitude to highlight the zone where the acceleration is higher and compared with the corresponding picture in the experiments. The comparison shows that the simulations can accurately predict the drop geometries observed in the experiment, with an error on the time needed for breakup of about 5.3%.

**Figure 7:**
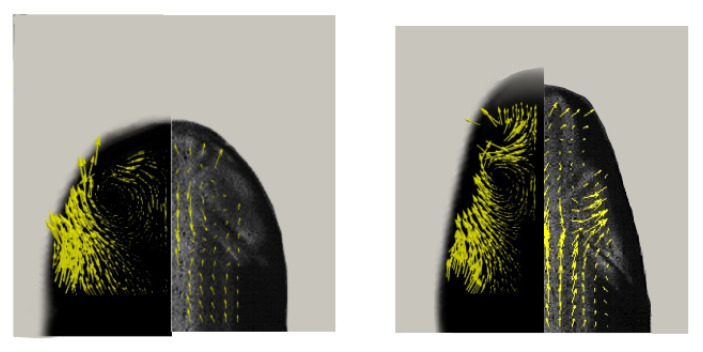
Comparison between the velocity vectors obtained by the numerical simulation (**left**) and the velocity vectors obtained by the micro-PIV analysis (**right**) in different instants. Both experimentally and numerically, it is possible to observe the formation of vortices within the discrete phase during the growth of the drop.

Finally, a comparison between the experimental and simulated velocity field in the mid-plane of the dispersed phase was performed. Figure 7 shows the velocity vector map obtained by the numerical simulations (left), together with the velocity vector map obtained by micro-PIV analysis (right), which has been mirrored. The vectors show a vortex in the thread nose, which is well known for droplets moving in two-phase flows [10]. The position and the dimensions of the vortexes obtained by the numerical simulation are in agreement with those obtained by the micro-PIV measurements. Furthermore, the velocity magnitude of the analysed fields is in agreement. In summary, we can conclude that the numerical simulations are in good agreement with the experiments both for the prediction of the dynamics of the droplet formation and for the velocity distribution.

### 3.2. Interface Dynamics during Droplet Breakup

**Figure 8:**
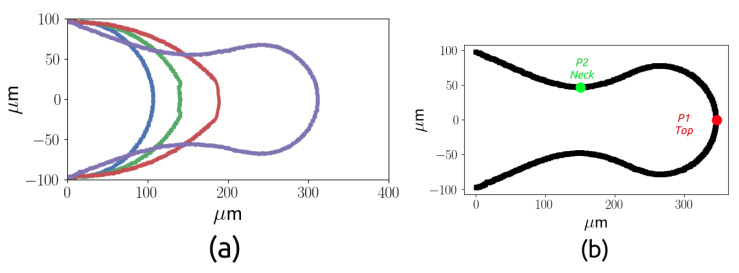
Thread interface at four time instants with δt=0.0004 s (**a**) and final thread interface (**b**), for Q* = 0.08.

The dynamics of the droplet during the breakup is analysed in this section, starting from the case with a dimensionless flow rate $Q^{*}$, defined as $Q^{*}=\frac{Q_{d}}{Q_{c}}$, equal to 0.08 and $Ca_{c}=6.7\times 10^{-3}$ and increasing the dispersed phase flow rate. During the breakup, three stages can be observed. In a first phase, called the filling stage, the dispersed phase evolves as a thread which starts with a hemispherical shape, then the thread increases with the shape of a cylinder with a rounded front end which we will call the thread nose. In the second phase, called necking, the thread starts to be squeezed, while the thread nose position moves along the junction. The final phase is the pinch-off, when the neck thickness decreases quickly until the thread surface breaks and the droplet is created. Figure 8 shows the thread interface at four time instants equally spaced from t=0 to t=0.0012 s, during the first two phases.

**Figure 9:**
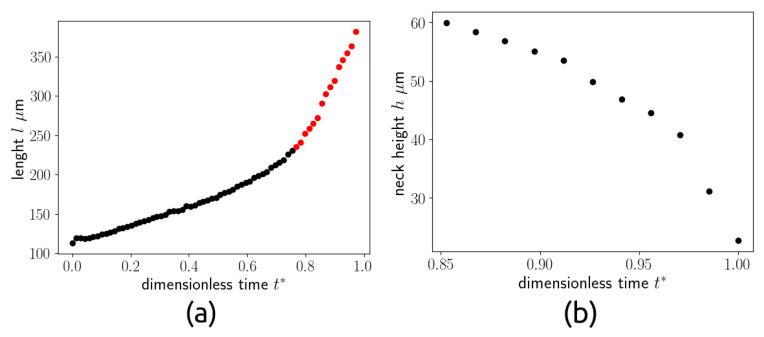
Evolution of the length of the evolving droplet in time (**a**) and evolution of the neck thickness (**b**) for Q* = 0.08.

The thread at the final instant is also shown in the figure (b) to show the point P1, which represents the position of the thread nose, and the point P2, which represents the position of the neck. Figure 9 shows the evolution in time of P1 (a) and the evolution in time of the thickness of the neck P2 (b) as a function of the dimensionless time.

**Figure 10:**
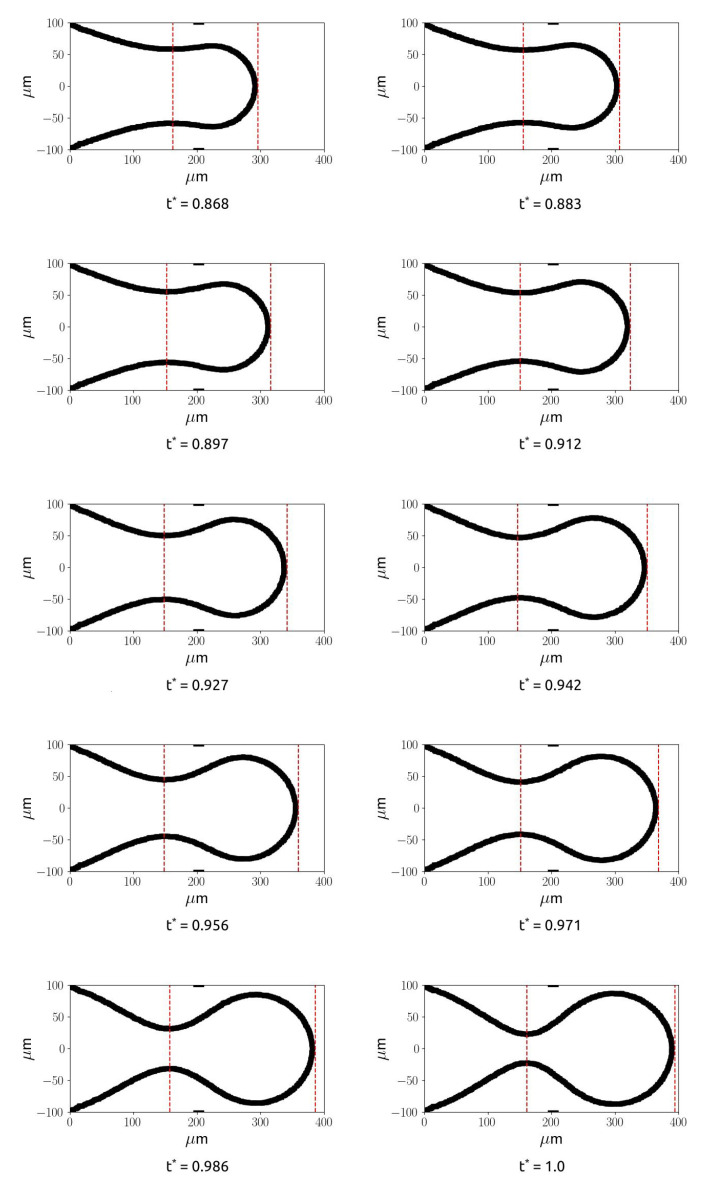
Evolution of the droplet with time. The two red lines refer to the position of point P1 and point P2, and the two black lines represent the position of the restriction in the junction, for Q* = 0.08.

The dimensionless time has been obtained by dividing the time by the time interval between a droplet detachment and the successive droplet detachment, equal to 16.6 ms for the considered case (Q* = 0.08)). Figure 9a shows that the filling and necking phases last for most of the total time, while P1 increases slowly until $t^{*}=0.85$ (black dots), while for $t^{*}>0.85$, it increases faster. Figure 9b also shows that the neck thickness decreases slowly for $t^{*}<0.9$ and drops very quickly for $t^{*}>0.95$. The first time interval corresponds to the filling and necking phases, while the second one characterises the pinch-off stage. Figure 10 shows the evolution of the thread interface with time, from the first instant where the neck starts to be visible until the last time instant before the breakup. The dimensionless time has been obtained in this case dividing by the time duration of the necking stage.

**Figure 11:**
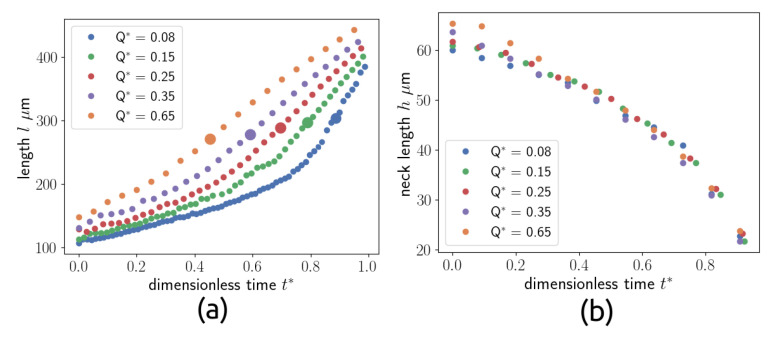
Evolution of the length of the thread (point P1) (**a**) and neck thickness (point P2) (**b**) versus time for flow rates. The detachment times used to evaluate the dimensionless time are [16.6, 10.4, 7.2, 5.4, 4.0] ms going from Q* = 0.08 to Q* = 0.65.

The figure shows that during the necking phase, the squeezing of the thread in the neck is slow, and the diameter of the thread can be assumed as a constant for a large time interval before the pinch-off phase. Similar trends can be shown at different flow rate ratios, as shown in Figure 11.

**Figure 12:**
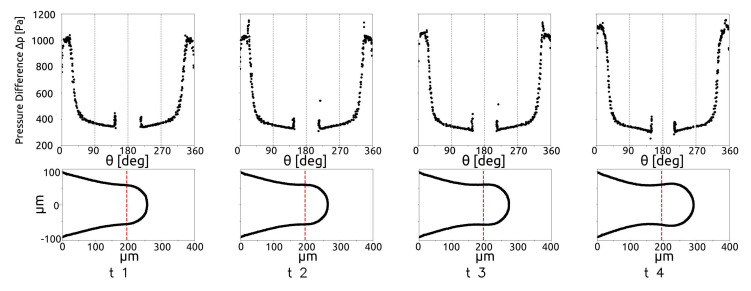

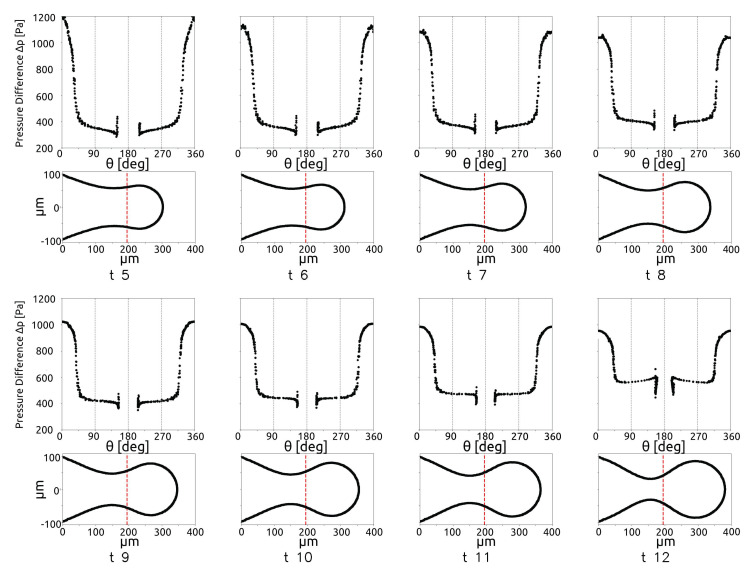
Pressure difference between the phases along the droplet surface during the breakup, for Q* = 0.08.

In Figure 11, the evolution of P1 and P2 are shown for $Q^{*}$ = [0.08, 0.15, 0.25, 0.35, 0.65]. The figure shows that P1 increases proportionally to $Q^{*}$, in the necking phase, while the neck thickness P2 does not depend on $Q^{*}$, i.e., the neck collapses with the same trend in all the cases. This can suggest that the duration of the breakup is a key parameter of the droplet creation process. During the breakup phenomena, the main forces acting on the drop are shear-stress at the interface and the pressure differences between the two phases. In the squeezing regime, the effect of the pressure build-up is the most important [12], because the thread can occlude the micro-channel in the junction, then the pressure in the continuous phase increases, breaking the interface. To investigate this phenomena, the pressure difference between the phases is plotted along the interface during the process, as shown in Figure 12. The plot is made in polar coordinates, where the angle 0 is on the thread nose and the angle 180 is on the inlet section.

**Figure 13:**
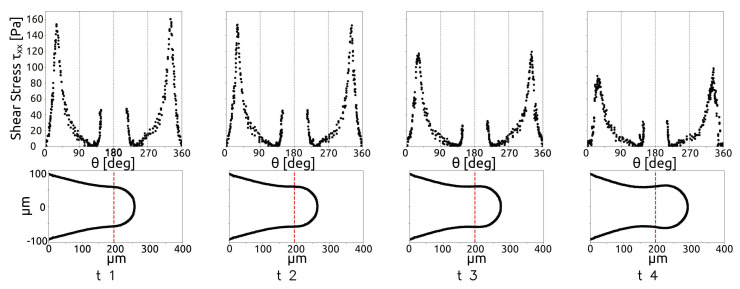

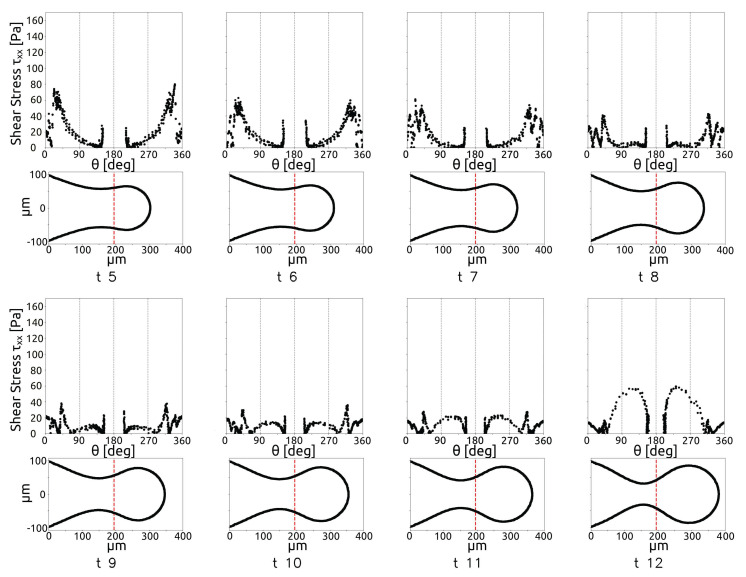
Shear-stress evolution along the drop during the breakup, for Q* = 0.08.

In Figure 12, the red dashed lines indicate the position of the restriction in the micro-junction. The figure shows that, when the thread occupies a large portion of the channel, the pressure difference increases, while it decreases when the forming drop has already crossed the restriction in the junction. In a similar way, it is possible to describe the shear-stress acting on the thread surface, as shown by Figure 13 for the same time instant as those shown in Figure 12.

Figure 13 shows that the shear is higher when the thread occupies the restriction in the first time instants, as the continuous flow rate reaches the maximum velocity due to the reduction in the cross section. Then, the shear decreases when the cross-section free for continuous phase passage increases. Figure 12 and Figure 13 show that the shear stress is lower than the pressure difference between the phases for all the time instants considered.

## 4. Discussion

The length of the droplets obtained by the simulations in the function of the flow rate ratio are reported in Table 2. In the table, ${\dot{Q}}^{*}$ indicates the flow rate ratio, and $l^{*}=l/W_{j}$ is the dimensionless length of the droplet.

Many papers in the literature show a linear correlation between $l^{*}$ and ${\dot{Q}}^{*}$ for T-junctions [4,22,23] and cross-junctions [24]. By fitting the dimensionless length obtained by the simulations, we obtain the following relation

(8) $$\frac{l}{w_{j}}=\alpha Q^{*}+\beta =0.382Q^{*}+0.905$$

with an R^2^ = 0.98 and an average error between the linear fit and the simulations of about 1.3%.

**Figure 14:**
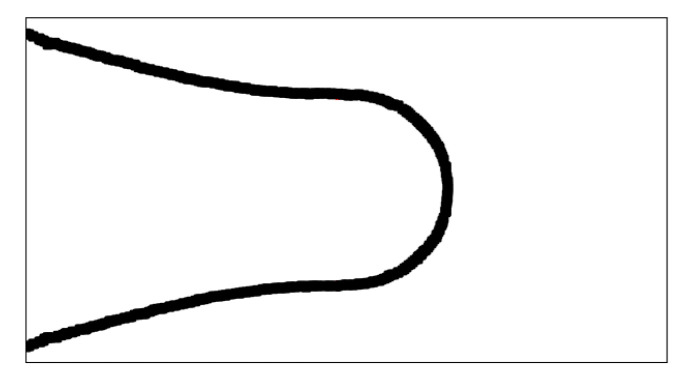
Control volume considered for the energy balance.

In order to find a model which supports this trend, the dynamics of the droplet formation can be considered, following the approach shown in [25] for predicting the time interval which involves the necking stage. The approach is applied to the entire necking stage, as it affects the final droplet size more than the filling stage, as shown by [26]. The control volume considered in this approach is shown in Figure 14.

The control volume is a cylinder containing the volume of the thread at the beginning of the necking stage, as shown in the figure. This region is inside the restriction in the junction. The final droplet volume is assumed to be given by the product of the dispersed phase volume flow rate multiplied by the time lapse between the beginning of the necking phase and the pinch-off. The droplets created in the junction show a circular shape by the high-speed camera images which record the the droplets on a plane xy containing the direction of the flow, as shown in Figure 5. This means that the volume of the created droplet is a sphere in the case a droplet diameter (that we will call the length of the droplet $l_{d}$) smaller than the height of the channel *H* and is a cylinder if $l_{d}>H$. Then, the volume of the droplet is $V_{d}=4/3\pi r_{d}^{3}$ if $l_{d}<H$, or $V_{d}=\pi r_{d}^{2}H$ if $l_{d}>H$. Assuming that the time interval between the beginning of the droplet creation and the time instant when the droplet detaches from the thread is δt, the volume of the droplet is

(9) $$V_{d}=V_{0}+{\dot{V}}_{d}\delta t=\frac{\pi l_{d}^{2}H}{4}$$

where ${\dot{V}}_{d}$ is the dispersed volume flow rate, and $V_{0}$ is the volume of the thread at the end of the filling stage, i.e., at the beginning of the necking stage, which is the stage described by the modelling in this section. Then, the length of the droplet $l_{d}$ after the detachment can be obtained by the dispersed phase volume flow rate,

(10) $$l_{d}={\left(\frac{4(V_{0}+{\dot{V}}_{d}\delta t)}{\pi H}\right)}^{1/2}$$

The time interval for the droplet creation during the necking stage can be obtained by the energy balance approach introduced by [25]. The surface tension energy stored in the thread before the droplet detachment is balanced by the energy difference between the inlet and the outlet sections in the control volume after the droplet detachment,

(11) $$\sigma S_{d}=M_{d}\left(\frac{pc}{\rho_{d}}+\frac{u_{d}^{2}}{2}-\frac{p_{d}}{\rho_{d}}\right)$$

where σ is the surface tension between the two phases, $S_{d}=2\pi rl$ is the surface of the droplet in the region of the neck approximated by a cylinder with radius *r* and length *l*, and $M_{d}=\pi r^{2}l\rho_{D}$ is its mass. If $p_{c}$ and $p_{d}$ are the pressure in the continuous and dispersed phase, respectively, and $u_{d}$ is the velocity of the droplet after the detachment, on the right of the equation, we have the energy of the droplet after creation. Equation (11) can be rewritten as

(12) $$\frac{u_{d}^{2}}{2}=\frac{2\sigma }{r\rho_{d}}+\frac{p_{d}-p_{c}}{\rho_{d}}$$

and considering that $\frac{p_{d}-p_{c}}{\rho_{d}}=\frac{\sigma }{\rho_{d}r}$, one obtains the velocity of the dispersed phase as follows:

(13) $$u_{d}={\left(\frac{6\sigma }{\rho_{d}r}\right)}^{1/2}$$

Assuming that $u_{d}=l/\delta t$, then one obtains

(14) $$\delta t=l{\left(\frac{\rho_{d}r}{6\sigma }\right)}^{1/2}$$

Substituting Equation (14) in Equation (10), one obtains

(15) $$l_{d}={\left(\frac{4}{\pi H}\left(V0+V_{d}l{\left(\frac{\rho_{d}r}{6\sigma }\right)}^{1/2}\right)\right)}^{1/2}$$

**Figure 15:**
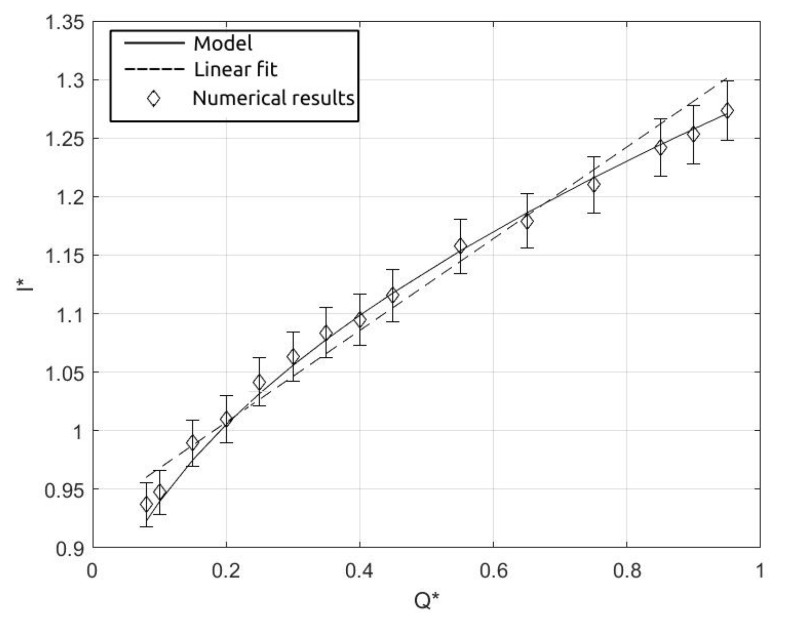
Dimensionless droplet length versus dimensionless dispersed volume flow rate. Symbols refer to experimental results, continuous line refers to Equation (16), and dashed line refers to Equation (8).

Dividing the droplet length by the width of the junction $W_{j}$ and rewriting the equation in a dimensionless form one obtains

(16) $$l^{*}=l_{0}^{*}+a\dot{(}Q^{*}{)}^{1/2}We_{c}^{1/4}$$

where $We_{c}=\frac{\rho_{c}v_{c}^{2}W_{j}}{\sigma }$ is the Weber number referred to the continuous phase in the junction, and $v_{c}=\frac{4{\dot{V}}_{c}}{\pi W_{j}^{2}}$ is the superficial velocity of the continuous phase in the junction. In the equation, $a={\left(\frac{l^{2}\rho_{d}}{6H^{2}\rho_{c}}\right)}^{1/4}$ is a constant with $l=4W_{j}$, and $l_{0}^{*}=0.8$ is obtained by the initial volume of the thread. Equation (16) is in very good agreement with the measurements, as shown by Figure 15.

In the figure, the dimensionless length of the droplets obtained by the numerical simulations is shown by the symbols, while the continuous line refers to the results obtained by Equation (16), and the dashed line represents the linear fit described in Equation (8). The error between the numerical results and Equation (16) is less than 1%, showing a better fit with respect to the linear correlation, where the average error was around 1.3%. This result has been obtained on the basis of an energy balance on the emerging dispersed phase entering the continuous phase channel, accordingly with [25]. The result shows a dependence of the droplet dimensions with the volume flow ratio and on the Weber number. A similar dependence on the We number has been observed also by in T-junctions [27]. Here, the dependence on the Weber number evaluated in the junction emphasises the influence of the restriction of the junction which forces the continuous phase to flow at a higher velocity in the region near the neck. These results can be a basis for the optimal design of micro cross-junctions with restriction and flow-focusing devices for the production of drops with targeted diameters in the function of the inlet flow rates.

## 5. Conclusions

The dynamics of the droplet formation in a micro cross-junction with a restriction has been studied by an integrated approach between CFD simulations in the OpenFOAM environment, experimental measurements, and an up-scaled model of the droplet breakup. The results of the simulations have been validated by a comparison with experimental measurements by means of high-speed camera images of the droplets and velocity measurements in the dispersed phase by micro-PIV. Through numerical simulations, the forces acting on the dispersed phase during the droplet formation and the droplets diameters have been obtained for different dimensionless flow rates. These results have been used for the development of a model of the droplet breakup under the squeezing regime and a novel correlation between the dimensionless length of the droplet, the dimensionless flow rate, and the We number. This correlation is more accurate then the linear one which is usually found in the literature. The methodology introduced in this paper can be used for the optimisation and control of droplet production in microfluidics applications.

## Figures and Tables

**Table 1:** Comparison of the drop length obtained from the numerical simulations (NS) and from the experimental measures (EXP).

Q*	Drop Length NS [µm]	Drop Length EXP [µm]	Error [%]
0.08	178	171	4.0
0.15	188	191	1.6
0.25	198	203	2.9
0.35	206	216	5.0
0.65	224	232	3.1

**Table 2:** Drop length in function of $Q^{*}$.

Sim.	Q*	Drop Length *l* [µm]	$l/w_{j}$
1	0.08	178	0.913
2	0.10	180	0.923
3	0.15	188	0.964
4	0.20	192	0.984
5	0.25	198	1.015
6	0.30	202	1.036
7	0.35	206	1.056
8	0.40	208	1.067
9	0.45	212	1.087
10	0.55	222	1.128
11	0.65	224	1.149
12	0.75	230	1.179
13	0.85	236	1.210
14	0.90	238	1.221
15	0.95	242	1.241

## Data Availability

Dataset available on request from the authors.

## Abbreviations

The following abbreviations are used in this manuscript:

CFD	Computational fluid dynamics
PIV	Particle image velocimetry
